# Preventive Effect of Curcumin Against Chemotherapy-Induced Side-Effects

**DOI:** 10.3389/fphar.2018.01374

**Published:** 2018-11-27

**Authors:** Zhijun Liu, Pengyun Huang, Siukan Law, Haiyan Tian, Wingnang Leung, Chuanshan Xu

**Affiliations:** ^1^Key Laboratory of Molecular Target and Clinical Pharmacology, State Key Laboratory of Respiratory Disease, School of Pharmaceutical Sciences, Fifth Affiliated Hospital, Guangzhou Medical University, Guangzhou, China; ^2^Faculty of Medicine, School of Chinese Medicine, The Chinese University of Hong Kong, Shatin, Hong Kong; ^3^Shenzhen Research Institute, The Chinese University of Hong Kong, Shenzhen, Shenzhen, China; ^4^Institute of Traditional Chinese Medicine and Natural Products, College of Pharmacy, Jinan University, Guangzhou, China; ^5^Division of Chinese Medicine, School of Professional and Continuing Education, The University of Hong Kong, Pokfulam, Hong Kong

**Keywords:** curcumin, chemotherapy, side-effects, cancer, natural products

## Abstract

Cancer is still a severe threat to the health of people worldwide. Chemotherapy is one of main therapeutic approaches to combat cancer. However, chemotherapy only has a limited success with severe side effects, especially causing damage to normal tissues such as bone marrow, gastrointestine, heart, liver, renal, neuron, and auditory tissues, etc. The side-effects limit clinical outcome of chemotherapy and lower patients’ quality of life, and even make many patients discontinue the chemotherapy. Thus, there is a need to explore effective adjuvant strategies to prevent and reduce the chemotherapy-induced side effects. Naturally occurring products provide a rich source for exploring effective adjuvant agents to prevent and reduce the side effects in anticancer chemotherapy. Curcumin is an active compound from natural plant *Curcuma longa* L., which is widely used as a coloring and flavoring agent in food industry and a herbal medicine in Asian countries for thousands of years to treat vomiting, headache, diarrhea, etc. Modern pharmacological studies have revealed that curcumin has strong antioxidative, anti-microbial, anti-inflammatory and anticancer activities. Growing evidence shows that curcumin is able to prevent carcinogenesis, sensitize cancer cells to chemotherapy, and protect normal cells from chemotherapy-induced damages. In the present article, we review the preventive effect of curcumin against chemotherapy-induced myelosuppression, gastrointestinal toxicity, cardiotoxicity, hepatotoxicity, nephrotoxicity, neurotoxicity, ototoxicity, and genotoxicity, and discuss its action mechanisms.

## Introduction

Cancer still a severe threat to the health of human is beings. The WHO recent report shows that cancer has become the second leading cause of death worldwide, almost 1 in 6 deaths are due to cancer in 2015 (World Health Organization [WHO], 2018). It is estimated that the new cases of cancer patients will rise rapidly in recent years, the morbidity is expected to reach approximately 24 million in 2035 (Huang, 2006). Not only does cancer bring serious pains to patients, lower their life quality, and also carries a significant economic burden to their families and governments. Therefore, cancer has been a severe public health problem worldwide.

Chemotherapy is one of the commonest therapeutic modalities in the management of cancer. However, the drugs currently used in the chemotherapy only have a limited success with severe side-effects, including myelosuppression, gastrointestinal toxicity, cardiotoxicity, hepatotoxicity, neurotoxicity, ototoxicity, etc. And then these serious side-effects make many patients discontinue the chemotherapy (Zhou et al., 2011; Salehi et al., 2014; Irving et al., 2015). Thus, there is a need to explore effective adjuvant strategies to prevent and reduce the chemotherapy-induced side effects.

**FIGURE 1:**
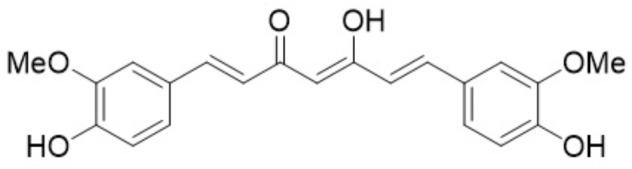
Curcumin formula.

Naturally occurring products as adjuvant therapy have been shown a promising potential in preventing the chemotherapy-induced side effects (Palipoch et al., 2014). Curcumin (C_21_H_20_O_6_) (Figure 1) is an active compound from natural plant *Curcuma longa* L., which is widely used as a coloring and flavoring agent in food industry and a herbal medicine in Asian countries for thousands of years to treat vomiting, headache, diarrhea, etc. Recently, pharmacological studies have revealed that curcumin has strong antioxidative, anti-microbial, anti-inflammatory and anticancer activities (Bishnoi et al., 2011; Wang et al., 2011a,b, 2012; Shiau et al., 2012; Mendonça et al., 2013; Sankrityayan and Majumdar, 2016; Dai et al., 2018). Growing evidence shows that curcumin is a very safe product to human being (Shehzad et al., 2013). It can not only prevent carcinogenesis and enhance clinical efficacy of chemotherapy through sensitizing cancer cells to the commonly used chemotherapy, and also protect normal cells from chemotherapy-induced damages (Zhou et al., 2011; Mendonça et al., 2013). In the present article, we review the preventive effect of curcumin against chemotherapy-induced myelosuppression, gastrointestinal toxicity, cardiotoxicity, hepatotoxicity, nephrotoxicity, neurotoxicity, ototoxicity and genotoxicity, and discuss its action mechanisms. Our aim is to provide some reference information for researchers and scientists in basic and clinical settings.

## The Preventive Effects of Curcumin Against Chemotherapy-Induced Toxicity

### Curcumin Ameliorates Chemotherapy-Induced Gastrointestinal Toxicity

Gastrointestinal toxicity is one of the commonest chemotherapy-induced side-effects. The patients receiving chemotherapy almost suffer from vomiting, diarrhea, nausea and anorexia (Yao et al., 2013). A reported study showed that a third of patients receiving chemotherapeutic agent 5-FU have life-threatening diarrhea because 5-FU-induced gastrointestinal toxicity (Irving et al., 2015). Mucosal barrier injury, including an increased villous atrophy and elevated intestinal permeability in gastrointestinal tract, is an important adverse effect of anticancer drugs. van’t Land et al. (2004) found that the absorption surface of the duodenum were remarkably decreased to 38 ± 6% on day 3, and the absorption surface of jejunum further reduced on day 4 after chemotherapy. In the meantime, they also observed methotrexate (MTX)-induced intestinal damage with marked increase of MPO+ cell influx in the duodenum and the jejunum. Interestingly, in the animals pretreated with curcumin at 2.5 mg/kg starting 24 h prior to the first MTX administration, the authors observed a remarkable re-establishment of villous structure without weight loss and marked increase of MPO+ cell influx (van’t Land et al., 2004). Yao et al. (2013) also reported that curcumin could improve 5-FU-induced diarrhea, decease 5-FU-associated weight loss, attenuate 5-FU-induced mucosa atrophy and villi loss, and reverse 5-FU-induced dramatic increase of serum endotoxin D-lactate and D-amino-acid oxidase (DAO) (Wang et al., 2011b). These findings demonstrate that curcumin has protective effect on chemotherapy-induced intestinal dysfunction and mucosa morphology.

### Curcumin Reduces Chemotherapy-Induced Cardiotoxicity

Cardiotoxicity is a dose-limiting factor affecting the clinical outcome of chemotherapy. Growing evidence shows that chemotherapy-induced cardiotoxicity involves oxidative stress, mitochondrial damage, calcium flux alteration and activation of proapoptotic signaling cascades, etc. (Dayton et al., 2011). Doxorubicin (DOX) is a common drug used in the management of malignancies, however, the lethal cardiac side-effect limits the therapeutic efficacy of DOX, and even some patients have to discontinue the DOX therapy (Swamy et al., 2012). Swamy AV reported that repeated use of Dox (15 mg/kg) for 2 weeks induced cardiotoxicity with elevated cardiac toxicity markers serum creatine kinase (CK) and lactate dehydrogenase (LDH), and decreased antioxidant enzyme [superoxide dismutase (SOD), catalase (CAT), and glutathione peroxidase (GTP) activities]. Curcumin (200 mg/kg, po) was then used as pretreatment for 2 weeks and for another 2 weeks in combination with Dox. The results showed that curcumin administration remarkably reduced the elevated level of cardiac toxicity markers and protected myocardium from Dox damage (Swamy et al., 2012). Benzer et al. (2018) found that DOX-treated rats produced the elevated CK and LDH, and decreased SOD, CAT and GTP activities. Curcumin administration via oral routine (100 or 200 mg/kg body weight) for 7 days could significantly lowered serum CK and LDH, and enhanced the SOD, CAT, GTP activities (Benzer et al., 2018). Sheu et al. (2015) reported that pretreatment of curcumin (30 μM) increased the antioxidant ability of normal cells and attenuated DOX-associated cardiotoxicity. Venkatesan N revealed that administration of curcumin (200 mg/kg) could significantly attenuate the early manifestation of adriamycin (ADR)-induced cardiotoxicity such as ST segment elevation and an increase in heart rate, and prevent the elevation in ADR-induced serum CK and LDH (Venkatesan, 1998). Bahadir et al. (2018) found that cisplatin caused severe myocardial degenerative alterations, a remarkable increase in malondialdehyde (MDA) level and significant decrease in CAT and SOD activities. However, the cisplatin-induced cardiotoxic manifestations markedly improved by the pretreatment of curcumin (Bahadir et al., 2018). The above evidence demonstrates that curcumin has protective activity against chemotherapy-induced cardiotoxicity.

### Curcumin Prevents Chemotherapy-Induced Hepatotoxicity

Hepatotoxicity is a well-described side effect of chemotherapy and is also a major limitation to its clinical application. Palipoch et al. (2014) found that cisplatin could significantly reduce hepatic SOD and CAT activities, and increase hepatic MDA levels and serum alanine aminotransferase (ALT) and aspartate aminotransferase (AST) levels. Histopathological observation showed that cisplatin induced remarkable hepatocytic damages such as liver congestion and ground glass changes. Pretreatment with curcumin and/or α-tocopherol could improve cisplatin-induced damages to hepatic enzymes and histopathology (Palipoch et al., 2014). Mohamad et al. (2009) reported that a single dose of DOX (7.5 mg/kg) could induce hepatotoxicity with serum ALT and AST elevations. The pretreatment of *Curcuma longa* L. extract could significantly restore the ALT and AST level, and prevent hepatotoxicity from DOX. Fetoni et al. (2014) found that the dose of 200 mg/kg curcumin not only has no toxic effects on the treated animal liver, and also attenuate DOX-induced hepatotoxicity. Waseem et al. (2017) revealed that oxaliplatin (200 μg/mL), a platinum-based chemotherapeutic agent, significantly increased lipid peroxidation (LPO) levels and protein carbonyl (PC) contents, and reduced the levels of glutathione (GSH) and non-protein thiol (NP-SH) in the isolated rat liver mitochondria. Oxaliplatin was also found to lower antioxidant and respiratory chain enzymes activities in mitochondria. The pretreatment of curcumin (5 μM) markedly restored the levels of LPO, GSH, NP-SH, and PC contents, and antioxidant and mitochondrial respiratory chain enzymes activities (Waseem et al., 2017). They also found that pretreatment of curcumin prior to cisplatin could prevent cisplatin-induced hepatotoxicity in rat model (Waseem et al., 2014). Wang et al. (2014) investigated the effect of curcumin on cisplatin-induced hepatotoxicity and ultrastructural damage. Their results showed that cisplatin had significant liver damage with vacuolated cytoplasm and blurred trabecular structure after cisplatin (50 mg/kg/day) was injected via i.p to Kunming mice for 10 days, and curcumin treatment (200 mg/kg/day) for 10 days could prevent cisplatin-induced liver damage (Wang et al., 2014). Hemeida and Mohafez (2008) found that a single dose of MTX (20 mg/kg I.P.) was able to induced hepatotoxicity with mild inflammation and necrosis in hepatocytes and sinusoidal cells as well as decreased liver SOD and CAT activities, and increased MDA level. Curcumin treatment (100 mg/kg, I.P.) once daily for 5 days after MTX injection restored liver SOD and CAT activities, and MDA level, and ameliorated MTX-induced liver damage (Hemeida and Mohafez, 2008).

### Curcumin Ameliorates Chemotherapy-Induced Nephrotoxicity

Nephrotoxicity is a severe chemotherapy-associated side-effect. Chemotherapy often causes kidney injury through damaging renal structure and function (Zhou et al., 2011). For example, cisplatin increases the levels of creatinine (Cr) and BUN, and causes directly swelling of proximal tubular cells with cytoplasmic vacuolization and necrosis (Ortega-Domínguez et al., 2017). Ortega-Domínguez et al. (2017) found that cisplatin-associated nephrotoxicity could be prevented by curcumin treatment. The kidney in the combined treatment group with curcumin and cisplatin showed minimal cytoplasmic vacuolization without necrosis, and animals treated by curcumin showed no histological abnormality in the kidney. Transmission electronic microscopy showed that cisplatin-induced extensive mitochondrial abnormalities, including mitochondrial swelling and rupture of cristae, were significantly attenuated by curcumin treatment (Ortega-Domínguez et al., 2017). Experimental results from Zhou et al. (2011) showed that chemotherapeutic drug mitomycin (MMC) dose-dependently increased the levels of Cr and BUN in mice, suggesting that MMC induced severe damage to the kidney. After administration of curcumin the levels of Cr and BUN were brought back to those of the control. The abnormalities were less evident in the combined group of curcumin and cisplatin treatment, clearly indicating an efficient activity of curcumin in the prevention of these mitochondrial abnormalities (Zhou et al., 2011). Their results further confirm that curcumin reduce chemotherapy-associated nephrotoxicity.

### Curcumin Decreases Chemotherapy-Induced Ototoxicity

Ototoxicity is an unwanted side-effect seen in cancer patients received chemotherapy, especially, platinum-based chemotherapeutic agents. After platinum-based chemotherapy such as cisplatin treatment, approximately 60 to 80% of the patients suffer from bilateral, symmetric middle- or high-frequency hearing loss, which affects patients’ communication and impairs their quality of life (Salehi et al., 2014). Growing evidence shows that the increase of reactive oxygen species (ROS) is an important cause of cisplatin-induced ototoxicity. The use of antioxidants to balance the redox condition is a major strategy to protect or rescue the auditory function from cisplatin-induced ototoxicity. Curcumin as a common antioxidant agent was stands out as an important component to ameliorate chemotherapy-induced ototoxicity (Fetoni et al., 2014). Fetoni et al. (2014) found that cisplatin significantly enhanced lipid peroxidation expression in outer hair cells (OHC) and induced the OHC loss. And the use of curcumin (200 mg/kg) remarkably reduced lipid peroxidation expression and the OHC loss. This preclinical study showed that curcumin (200 mg/kg) treatment reduced hear loss of about 20 dB in the threshold compared to cisplatin treatment, indicating that curcumin ameliorated the onset of cisplatin-induced ototoxicity (Fetoni et al., 2014). Salehi et al. (2014) also found that cisplatin treatment could lead to an average hearing loss of 50 dB and a curcumin and dexamethasone-loaded nanoparticle could attenuate cisplatin-induced hearing loss across all of the hearing frequencies.

### Curcumin Attenuates Chemotherapy-Induced Myelosuppression

Chemotherapeutic agents frequently damage bone borrow cells, subsequently causing myelosuppression, which increases susceptibility to microbial infection. Chemotherapeutic agent Etoposide treatment (50 mg/kg b.w., intraperitoneally) for 3 days caused serious hypoplasia of bone marrow. And then curcumin (100 and 200 mg/kg b.w.) was administrated to the Etoposide-treated rats via gavage for 7 days. The results showed that curcumin improved significantly etoposide-induced the percentage of granulocytic precursors and lymphocytes, demonstrating that curcumin has a remarkable activity in attenuating chemotherapy-associated myelosuppression (Papiez, 2013). Recently, Chen et al. (2017) found in a tumor-bearing animal that curcumin could improve tumor-associated anemia and survival rate of chemotherapeutic carboplatin-treated mice. Histologic study showed a significant improvement in the myelosuppression of chemotherapy-treated mice (Chen et al., 2017).

### Curcumin Attenuates Chemotherapy-Induced Neurotoxicity

Neurotoxicity is also a common adverse effect of chemotherapy. Among chemotherapy-induced neurotoxicities, peripheral neuropathy is the commonest one. Actually, after a full course of cisplatin therapy, patients often suffer from severe sensory peripheral neuropathies with extreme pain and low life quality (Mendonça et al., 2013). Curcumin was proved to possess protective action on neuronal cell lines and neuronal tissues. In clinical settings, curcumin is used as a neuroprotective agent in the management of epilepsy, Alzheimers’ disease, and other neurodegenerative disorders (Mendonça et al., 2013). Bishnoi et al. (2011) reported that curcumin had a protective action on haloperidol-associated neurotoxicity. Pretreatment of curcumin could dose-dependently prevent the chronic haloperidol-induced behavioral, cellular, and neurochemical changes (Bishnoi et al., 2011). Dai et al. (2018) found that curcumin could reduce colistin-induced neurotoxicity in N2a cells. Curcumin was also found to reduce the histological changes of the dorsal root ganglia (DRG) and sciatic nerve in the animal model of sciatic nerve crush. For example, curcumin treatment could attenuate the decrease of total number, diameter, and area of the damaged sciatic nerve fibers (Bishnoi et al., 2011). To reduce chemotherapy-induced neurotoxicity, Mendonça et al. (2013) used curcumin in their study for investigating its effect on cisplatin-induced neurotoxicity in NGF-differentiated PC12 cells. The results showed that 1.0 ug/ml of curcumin had no effect on cisplatin-induced neurite outgrowth, however, 10 ug/ml of curcumin could significantly decrease cisplatin-induced inhibition of neurite outgrowth by up to 50%. They also found in Wistar rats that curcumin could reduce cisplatin-induced degeneration of nerve fiber of the sciatic nerve (Mendonça et al., 2013).

### Curcumin Prevents Chemotherapy-Induced Genotoxicity

Growing studies also show that chemotherapy could cause genotoxicity, which might induce secondary cancer (Said Salem et al., 2017). In the Said Salem et al. (2017) study, a single dose of either cisplatin (6.5 mg/kg) or methotrexate (10 mg/kg) was injected intraperitoneally to mice. Kidney and bone marrow cells obtained from cisplatin- or methotrexate-treated mice was employed to measure DNA damage using a Comet assay, and bone marrow cells also used to measure chromosome damage using a micronucleus assay. Their results showed that cisplatin or MTX alone treatment significant increased the percentage of micronucleated polychromatic erythrocytes (MNPCEs) and DNA strand breaks (DNA damage) of kidney and bone marrow cells. The oral administration of curcumin at the dosage of 60, 90, or 120 mg/kg for three consecutive days before either cisplatin or methotrexate treatment significantly decreased the incidence of cisplatin- and methotrexate-induced micronuclei and DNA damage (Said Salem et al., 2017). Chen et al. (2017) used a probe derived from curcumin to investigate the effect of curcumin on DNA repair pathway in mice bone marrow cells. The results showed that curcumin could attenuate carboplatin-induced DNA damage through initiating DNA repair pathway (Chen et al., 2017).

## Action Mechanisms

Curcumin is proven to have capability of enhancing the therapeutic efficacy of chemotherapy and protecting normal cells from chemotherapy-induced toxicity. However, the action mechanisms remain to be further clarified. Growing evidence shows the bioeffects of curcumin possibly through modulating a series of target molecules such as adhesion molecules, inflammatory factors, transcription and growth factors, apoptosis-related proteins, and some enzymes and kinases, etc. (Fetoni et al., 2014). A series of studies demonstrated that curcumin could inhibit pro-inflammatory and inflammatory factors such as NF-κB, COX, LO, STAT3, Xanthine oxidase and inducible nitric oxide synthase, to sensitize cancer cells to chemotherapy and reduce the chemotherapy-induced toxicity (Calabrese et al., 2003; Belcaro et al., 2014; Fetoni et al., 2014; Benzer et al., 2018; Dai et al., 2018). Banerjee et al. (2017) reported that curcumin increased the expression level of pro-apoptotic molecules such as BAK and BID, and decreased the expression levels of apoptotic molecules such as BCL-2, BCL-XL, and MCL-1 in the docetaxel-treated cancer cells. They also found that curcumin could synergize docetaxel to activate tumor suppressor gene p53 and inhibit phosphoinositide 3-kinase (PI3K)/AKT, epidermal growth factor receptor (EGFR), and human epidermal growth factor receptor type 2 (HER2) (Banerjee et al., 2017). Moreover, heme oxygenase-1 is an important modulator of cytoprotection. Fetoni et al. (2014) in a preclinical study found that curcumin increased the expression level of heme oxygenase-1 and attenuated cisplatin-associated ototoxicity, indicating that curcumin attenuated cisplatin-induced ototoxicity via the up-regulation of heme oxygenase-1 expression.

ROS production is regarded as an important effector in chemotherapy-induced toxicity. Clearance of ROS in normal tissues is proven to be a major strategy for preventing chemotherapy-induced toxicity. Curcumin as an intracellular ROS scavenger show promising in reducing chemotherapy-induced toxicity through increasing intracellular levels of antioxidants and inhibiting lipid peroxidation in normal tissues (Calabrese et al., 2003, 2008; Mohamad et al., 2009; Belcaro et al., 2014; Palipoch et al., 2014; Sankrityayan and Majumdar, 2016; Dai et al., 2018). Sheu et al. (2015) reported that curcumin pretreatment (30 uM) could enhance the antioxidant ability of normal cells by increasing SOD activity to reduce intracellular damage of DOX-produced ROS on normal cells (Sheu et al., 2015). Palipoch et al. (2014) found that curcumin pretreatment could decrease liver lipid peroxidation and NADPH oxidase expression in cisplatin-treated rats to prevent cisplatin-associated liver damage. Curcumin is also proven to protect mitochondria from chemotherapy-induced oxidative stress. The results from Ortega-Domínguez et al. (2017) showed that curcumin attenuated cisplatin-associated damage to mitochondria through decreasing cisplatin-induced ROS production in mitochondria and reducing the cisplatin-induced increase of NAD^+^-dependent deacetylase sirtuin-3 (SIRT3) and mitophagy associated proteins such as parkin and phosphatase as well as the increase of mitochondrial dynamic modulators such as mitochondrial fission 1 protein (FISI), optic atrophy 1 protein (OPA1), etc. Their finding also demonstrated that curcumin reduced chemotherapy-induced toxicity through the regulation of mitochondrial bioenergetics and redox balance (Ortega-Domínguez et al., 2017).

## Safety and Curcumin-Drug Interaction

Curcumin has been used as a coloring agent and food additive in Asian countries for centuries. The experience from daily life and evidence from *in vitro*, *in vivo* studies and clinical trials consistently confirm that curcumin is a safe and effective natural product for preventing and treating malignancies (Kusuhara et al., 2012; Liu et al., 2012; Adiwidjaja et al., 2017; Mantzorou et al., 2018). Kusuhara et al. (2012) clinical trial showed no curcumin-related toxicity in patients who were given curcumin (8 g/day) via oral administration for 3 months (Liu et al., 2012). A review by Soleimani et al. (2018) on acute toxicity, subacute toxicity and subchronic toxicity of curcumin from published literatures since 1992 to the end of 2016 and reproductive toxicity, mutagenicity, and genotoxicity of curcumin from the literatures since 2006 to the end of 2016 also showed that curcumin is non-toxic, non-mutagenic, and non-genotoxic. FDA has categorized curcumin as a “generally recognized as safe” substance (Soleimani et al., 2018). Therefore, curcumin has widely used in the complementary therapy of cancer patients through combining with chemotherapeutic drugs. Growing evidence shows that curcumin can enhance therapeutic efficacy of many chemotherapeutic agents such as cisplatin, 5-fluorouracil, vinca alkaloid, vinorelbine and gemcitabine etc., and reduce their side effects through their pharmacological interaction (Crooker et al., 2018). It is well-known that drug-drug interaction also includes pharmacokinetic interaction besides pharmacological interaction. The drug-metabolizing enzymes and drug transporters are important factors affecting drug absorption, distribution, metabolism and excretion via pharmacokinetic interaction (Vilson and Maulik, 2018). Among them, cytochrome 450 (CYPs) and uridine dinucleotide phosphate (UDP)-glucuronosyltransferases (UGTs) are two major drug-metabolizing enzymes. Curcumin was found to have a competitive inhibition on CYPs, especially CYP1A2, CYP3A4, and non-competitive inhibition on CYP2D6 and CYP2C9 (De Paz-Campos et al., 2014; Koe et al., 2014; Devi et al., 2015; Adiwidjaja et al., 2017; Bahramsoltani et al., 2017; Zhou et al., 2017; Vilson and Maulik, 2018). Liu et al. (2012) demonstrated that curcumin also had ability to inhibit 10 members of UGTs including UGT1A1 and 2B7. The inhibitory effect of curcumin on CYPs and UGTs make it have ability to reduce the degradation of CYP substrates and clearance of UGT substrates. Recent studies also reveal that curcumin can inhibit drug transporters including P-glycoprotein (P-gp), multi-drug resistant associate protein (MRP), breast cancer resistance protein (BCRP), adenosine triphosphate-binding cassette (ABC) such as ABCG1, ABCG2, and ABCB1, organic anion-transporting polypeptide (OATP) such as OATP1B1 and OATP1B3, subsequently resulting in the absorption increase of the above transporter substrates (Liu et al., 2012; Devi et al., 2015; Zhou et al., 2017; Dogra et al., 2018; Wei et al., 2018). The above findings demonstrate that curcumin can change pharmacokinetic profile of the enzyme and transporter substrate drugs and increase their plasma levels through the inhibition of these drug-metabolizing enzymes and drug transporters. Of course, the increase of plasma level of chemotherapeutic drugs can directly enhance therapeutic efficacy. However, it should be kept in mind that physicians and patients should adjust or reduce the used dosage of the chemotherapeutic drugs, which are the substrates of the above enzymes and transporters, with narrow therapeutic window when they are co-administered with curcumin because curcumin has ability to increase their absorption and reduce the clearance.

## Conclusion

Curcumin is a safe natural product with anticancer, anti-inflammatory and antioxidant activities. It has recently received increased attention in protecting normal tissues from chemotherapy-induced toxicity. Curcumin can enhance therapeutic efficacy of many anticancer drugs and reduce their side effects through pharmacological and pharmacokinetic interactions. Growing evidence show that curcumin can reduce chemotherapy-induced toxicity through clearing intracellular ROS in normal tissues and modulating a series of target molecules such as adhesion molecules, inflammatory factors, transcription and growth factors, apoptosis-related proteins, and some enzymes and kinases, etc. (Calabrese et al., 2003, 2008; Mohamad et al., 2009; Belcaro et al., 2014; Fetoni et al., 2014; Palipoch et al., 2014; Sheu et al., 2015; Sankrityayan and Majumdar, 2016; Banerjee et al., 2017; Ortega-Domínguez et al., 2017; Benzer et al., 2018; Dai et al., 2018). However, the current evidence is mainly from the *in vitro* and *in vivo* animal experiments, few clinical trials have yet investigated the protective effect of curcumin against chemotherapy-induced toxicity (Mohajeri and Sahebkar, 2018). Thus, more clinical trials should be conducted in the future investigations to further confirm the prevention of curcumin on chemotherapy-induced toxicity. The recent studies have revealed that the major reason to limit clinical trial and application of curcumin is its instability, poor absorption, and rapid systemic elimination. The poor absorption and rapid clearance make curcumin have very low bioavailability and short retention time, subsequently resulting in very low plasma level (Belcaro et al., 2014; Salehi et al., 2014; de Oliveira et al., 2016; Sankrityayan and Majumdar, 2016). More recently, the improvement of bioavailability and retention time of curcumin has become a hotspot for clinical translation of curcumin. Nanoformulation provides an effective way to improve bioavailability and retention time of curcumin through enhancing its solubility, stability and absorption. Up to now, some inorganic and organic materials including iron oxide, silicon dioxide, ferritin, albumin, chitosan, oligosaccharides, cyclodextrin (CD), PAMAM dendrimers, poly (lactide-co-glycolide) (PLGA), poly(alkyl cyanoacrylate) (PACA), d-α-tocopheryl polyethylene glycol 1000-block-poly (β-amino ester) (TPGS-PAE), and monomethoxy poly(ethylene glycol)-poly(lactide) copolymers (MPEG-PLAs) have been used to fabricate curcumin nanoformulations (Acharya and Sahoo, 2011; Cui et al., 2013; Dilnawaz and Sahoo, 2013; Mollazade et al., 2013; Chen et al., 2014; Fang et al., 2014; Lou et al., 2014; Sankar et al., 2014; Poorghorban et al., 2015; Scarano et al., 2015; Wang et al., 2015; Chen et al., 2016; de Oliveira et al., 2016; Ndong Ntoutoume et al., 2016; Pawar et al., 2016b; Zheng et al., 2016; Zhang et al., 2017; Yadav et al., 2018). These as-prepared curcumin nanoformulations have been proven to have enhanced anticancer efficacy as well as significant improvement in bioavailability and retention time (Pramanik et al., 2012; Zheng et al., 2016; Falconieri et al., 2017; Thulasidasan et al., 2017). Zhang et al. (2017) reported that a nanoparticle containing curcumin and DOX enhanced anticancer efficacy of DOX and reduced its systematic toxicity. Pramanik et al. (2012) found that curcumin nanoparticles attenuated DOX-induced bone marrow suppression and cardiotoxicity. Pawar et al. (2016b) prepared docetaxel (DTX) and curcumin co-encapsulated biodegradable nanoparticles and found that the nanoparticles exhibited enhanced anticancer efficacy with reduced toxicity. Sankar et al. (2015, 2016) used PLGA as a membrane material to prepare curcumin-loaded nanoparticles and reported that these curcumin nanoparticles reduced arsenic-induced genotoxicity, hepatotoxicity, nephrotoxicity, and neurotoxicity (Scarano et al., 2015). To enhance the targeted features of curcumin nanoparticles, folic acid, hyaluronan and, and Epithelial cell adhesion molecule aptamer have been used target molecules to modifying the curcumin nanoparticles and found that the targeted curcumin nanoparticles have a more significant improvement in the bioavailability and retention time, and in the increase of antitumor activity and attenuation of chemotherapy-induced toxicity, in comparison with free curcumin and the non-targeted curcumin nanoparticles (Li et al., 2014; Chen et al., 2016; Pawar et al., 2016a; Sankar et al., 2016; Thulasidasan et al., 2017). Although nanoformulations show a great promising in enhancing curcumin effects on the reduction of chemotherapy-induced side effects, the tedious and complicated preparation and uneasy control of quality limited the wide application of curcumin nanoformulations. Therefore, easily prepared and controlled curcumin nanoformulations are expected to be used in reducing chemotherapy-associated side effects in the near future.

## Author Contributions

ZL searched relative articles and drafted the manuscript. PH and SL assisted in searching relative articles and drafting the manuscript. HT and WL supervised the manuscript writing and revised the manuscript. CX created the outline of the manuscript, supervised the manuscript writing, and revised it.

## Conflict of Interest Statement

The authors declare that the research was conducted in the absence of any commercial or financial relationships that could be construed as a potential conflict of interest.
